# Global Sensitivity Analyses of the APSIM-Wheat Model at Different Soil Moisture Levels

**DOI:** 10.3390/plants14172608

**Published:** 2025-08-22

**Authors:** Ying Zhang, Pengrui Ai, Yingjie Ma, Qiuping Fu, Xiaopeng Ma

**Affiliations:** 1 College of Hydraulic and Civil Engineering, Xinjiang Agricultural University, Urumqi 830052, China; zying202303@163.com (Y.Z.); xj-myj@163.com (Y.M.); qiupingfu@xjau.edu.cn (Q.F.); 2 Xinjiang Water Conservancy Project Safety and Water Disaster Prevention Key Laboratory, Urumqi 830052, China; 3 Western Agricultural Research Center of Chinese Academy of Agricultural Sciences, Changji 831100, China; jsgg889@163.com; 4 Research Institute of Soil, Fertilizer and Agricultural Water Conservation, Xinjiang Academy of Agricultural Sciences, Urumqi 830052, China

**Keywords:** APSIM-wheat model, sensitivity analysis, Morris method, EFAST method

## Abstract

The APSIM (Agricultural Production Systems Simulator)-Wheat model has been widely used to simulate wheat growth, but the sensitivity characteristics of the model parameters at different soil moisture levels in arid regions are unknown. Based on 2023~2025 winter wheat field data from the Changji Experimental Site, Xinjiang, China, this study conducted a global sensitivity analysis of the APSIM-Wheat model using Morris and EFAST methods. Twenty-one selected parameters were perturbed at ±50% of their baseline values to quantify the sensitivity of the aboveground total dry matter (WAGT) and yield to parameter variations. Parameters exhibiting significant effects on yield were identified. The calibrated APSIM model performance was evaluated against field observations. The results indicated that the order of influential parameters varied slightly across different soil moisture levels. However, the WAGT output was notably sensitive to accumulated temperature from seedling to jointing stage (T1), accumulated temperature from the jointing to the flowering period (T2), accumulated temperature from grain filling to maturity (T4), and crop water demand (E1). Meanwhile, yield output showed greater sensitivity to number of grains per stem (G1), accumulated temperature from flowering to grain filling (T3), potential daily grain filling rate during the grain filling period (P1), extinction coefficient (K), T1, T2, T4, and E1. The sensitivity indices of different soil moisture levels under Morris and EFAST methods showed highly significant consistency. After optimization, the coefficient of determination (R^2^) was 0.877~0.974, the index of agreement (d-index) was 0.941~0.995, the root mean square error (RMSE) was 319.45~642.69 kg·ha^–1^, the mean absolute error (MAE) was 314.69~473.21 kg·ha^–1^, the residual standard deviation ratio (RSR) was 0.68~0.93, and the Nash–Sutcliffe efficiency (NSE) was 0.26~0.57, thereby enhancing the performance of the model. This study highlights the need for more careful calibration of these influential parameters to reduce the uncertainty associated with the model.

## 1. Introduction

Wheat is one of the most important food crops in China, with the northwest region accounting for 35.9% of the country’s total wheat production area [1]. Xinjiang is situated in the inland northwest region, where it faces persistent aridity and low annual rainfall levels. Water scarcity has emerged as an urgent challenge that must be addressed in agricultural production. Under conditions of agricultural water scarcity, implementing a rational irrigation system is crucial for developing water-saving agriculture and serves as one of the key measures to ensure high crop yield, quality, and efficiency [2]. Aboveground total dry matter (WAGT) and yield are crucial variables in wheat production that directly influence its economic returns [3]. Soil moisture content and an appropriate level for initiating irrigation are crucial factors influencing wheat growth and yield [4]. The scientific and rational regulation of these factors can effectively enhance grain production [5].

Traditional management decision-making practices alone are insufficient for effective wheat management. Employing crop models is necessary to overcome the limitations of field experimental methods [6]. Crop models have been extensively utilized in fields such as crop yield prediction, climate change assessment, crop growth management, and agricultural risk evaluation [7,8,9,10]. Their mechanistic and predictive capabilities have been continually improved, leading to successful applications in agricultural production and resource management. Their significance in government decision-making is increasingly recognized [11,12]. Notable crop models used internationally include DSSAT (Decision Support System for Agrotechnology Transfer) [13], WOFOST (World Food Studies) [14], and APSIM (Agricultural Production Systems Simulator) [15]. Yang et al. [16] demonstrated that under drought conditions, the DSSAT model exhibits greater sensitivity to nitrogen than to water. This is because its water module only considers the relationship between potential water uptake and actual transpiration, resulting in less accurate simulations of crop yield losses under varying water stress levels. Meanwhile, the WOFOST model demonstrates limited performance in simulating wheat growth under drought and water stress conditions [17]. In contrast, the APSIM model has demonstrated superior applicability and has been widely applied in the North China Plain, Australia, and several major wheat-producing countries in Europe [18,19,20]. The simulation results consistently exhibit high accuracies and reliability. However, previous research concerning the APSIM model has mainly concentrated on wheat-producing regions with favorable irrigation or rainfed conditions. In water-scarce dryland wheat cultivation systems, research on the application of this model remains insufficient, and its suitability and simulation performance require systematic evaluation and optimization [21].

When applying the APSIM-Wheat model to a specific region, a major challenge lies in determining its parameters. To date, the APSIM-Wheat model contains approximately 45 parameters (The APSIM-Wheat Module (7.5 R3008)), and calibrating these parameters is a time-consuming and labor-intensive [22,23]. Sensitivity analysis (SA) identifies parameters that significantly influence output variables [24]. By optimizing only these influential parameters, the efficiency of parameter optimization can be greatly improved [25]. The SA methods can be categorized into local and global methods. Local SA methods rely on baseline parameter values and tend to neglect the complex interactions among the parameters [26]. In contrast, global SA evaluates the effects of simultaneous changes in multiple parameters and their interactions on the model outcomes. This global SA can be divided into qualitative and quantitative analyses. Qualitative approaches focus on assessing the relative importance of each parameter by employing techniques such as the Morris screening method, multiple regression, and the Fourier amplitude sensitivity test (FAST). Among these, the Morris method [27] is widely used and highly effective for identifying influential parameters within a large set. Quantitative methods, such as the variance-based Sobol method and extended Fourier amplitude sensitivity test (EFAST), quantify the contribution of each parameter to the model results. Unlike the Sobol method, which calculates total and partial variances, the EFAST approach simplifies the computation of sensitivity indices [28]. Recent research has made considerable progress in utilizing EFAST to assess the influence of APSIM-Wheat model parameters on simulation results, thereby optimizing the parameter-testing process [29].

Therefore, the objective of this study was to conduct the SA of the APSIM-Wheat model at different soil moisture levels in the Xinjiang region of China using Morris and EFAST methods. Specifically, (1) the sensitivity of WAGT and yield to parameter changes was quantified, and (2) the parameters that significantly impacted yield were optimized while evaluating the performance of the APSIM-Wheat model. The results of this study may provide model users with effective methods to identify and optimize influential parameters, thereby reducing the uncertainty associated with the model.

## 2. Results

### 2.1. Analysis Using the Morris Method

#### 2.1.1. Morris Results for WAGT

**Figure 1:**
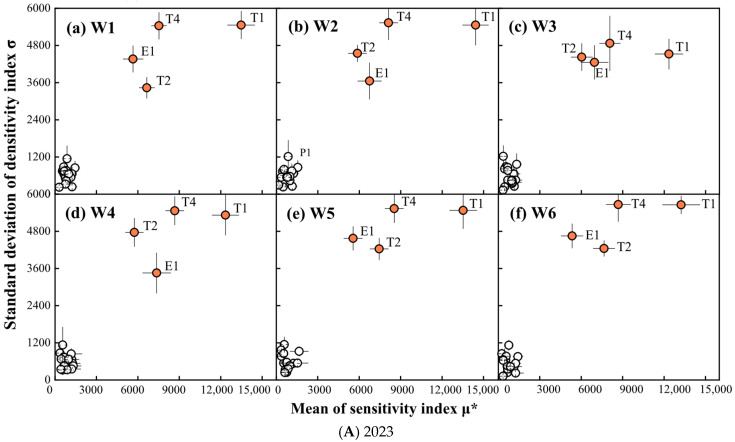

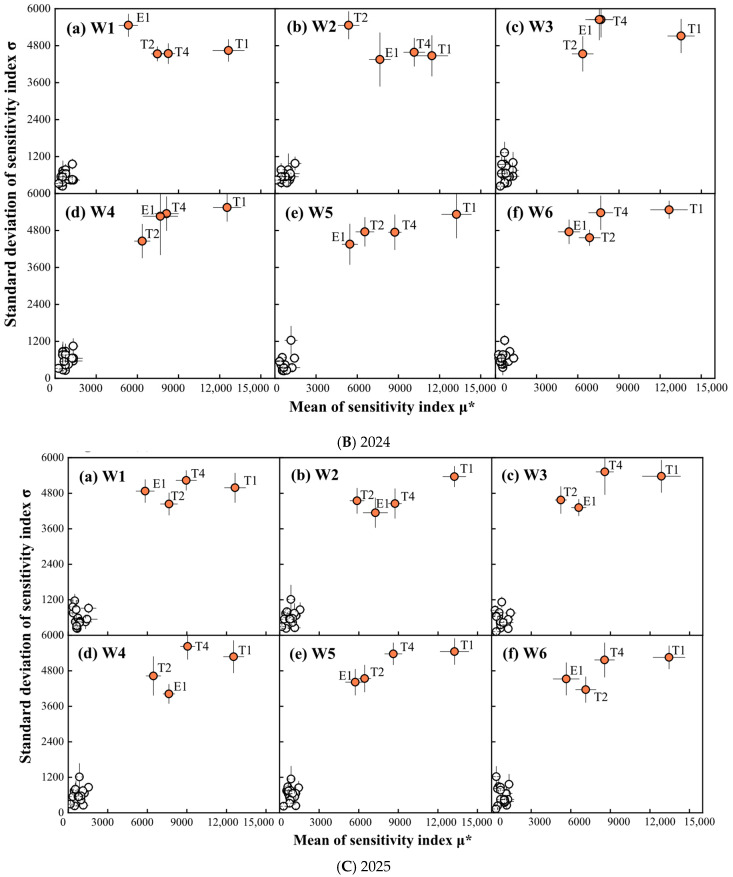
SA results of Morris method analysis on WAGT. W1: the soil moisture content (SMC) of 9.15% throughout the wheat season; W2: 13.07% SMC during heading and grain filling periods and 9.15% SMC during the other periods; W3: 5.23% SMC during heading and grain filling periods and 9.15% SMC during the other periods; W4: 5.23% SMC during greening and jointing periods and 9.15% SMC during the other periods; W5: 13.07% SMC during greening and jointing periods and 9.15% SMC during the other periods; W6: 13.07% SMC during greening, jointing, and heading periods and 9.15% SMC during the other periods.

The SA results associated with using the Morris method on the mean (μ*) and variance (σ) of WAGT are shown in Figure 1. The parameters that most significantly affected wheat WAGT output, in the order of their impact, were T1 (accumulated temperature from seedling to jointing stage), T4 (accumulated temperature from grain filling to maturity), and T2 (accumulated temperature from jointing to flowering period)/E1 (crop water demand). Under different water treatments, the sensitivity order of T2 and E1 varied, but T1 and T4 consistently remained among the top two. It indicated that T1 was the most significantly influential parameter, and it was greater than T2 and T4.

#### 2.1.2. Morris Results for Wheat Yield

**Figure 2:**
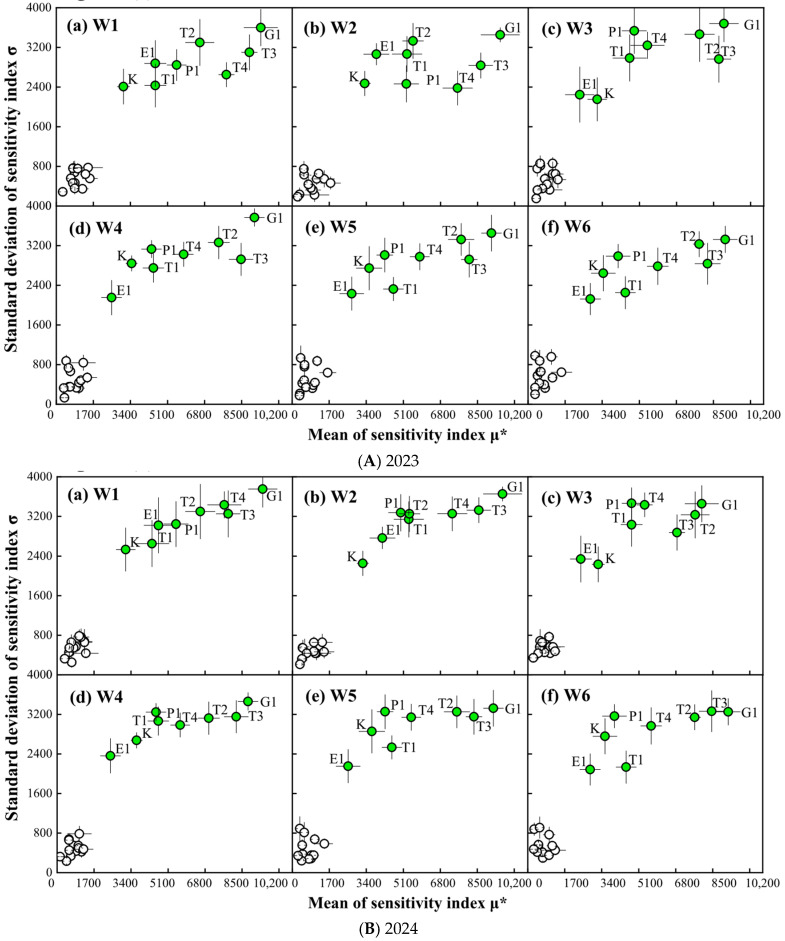

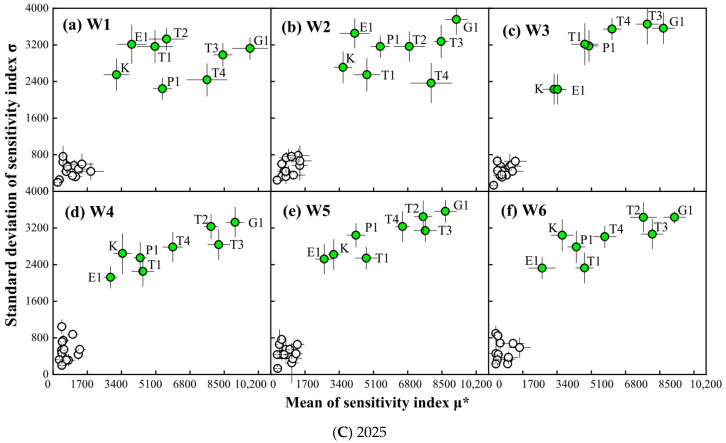
SA results of Morris method analysis on wheat yield. W1: the SMC of 9.15% throughout the wheat season; W2: 13.07% SMC during heading and grain filling periods and 9.15% SMC during the other periods; W3: 5.23% SMC during heading and grain filling periods and 9.15% SMC during the other periods; W4: 5.23% SMC during greening and jointing periods and 9.15% SMC during the other periods; W5: 13.07% SMC during greening and jointing periods and 9.15% SMC during the other periods; W6: 13.07% SMC during greening, jointing, and heading periods and 9.15% SMC during the other periods.

The SA results associated with using the Morris method on μ* and σ of the yield are shown in Figure 2. The SA results indicated that eight parameters were influential on wheat yield. Among these, G1 (number of grains per unit stem) and T3 (accumulated temperature from flowering to grain filling period) were the most influential parameters, with the largest μ* and σ, showing stronger interactions or nonlinear effects with other parameters. It was evident that T3 and G1 had a greater impact on the final yield. At different soil moisture levels, the influential parameters were the same, but the order of the four parameters P1 (potential daily grain filling rate during the grain filling period), T1, E1, and K (extinction coefficient) was slightly different.

### 2.2. Analysis Using the EFAST Method

#### 2.2.1. EFAST Results for WAGT

**Figure 3:**
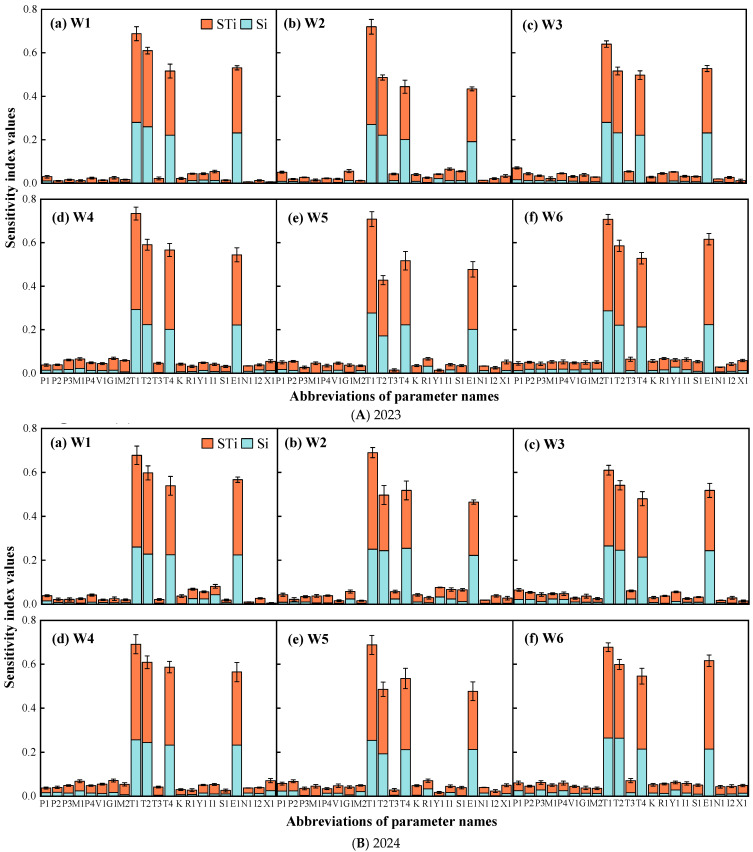

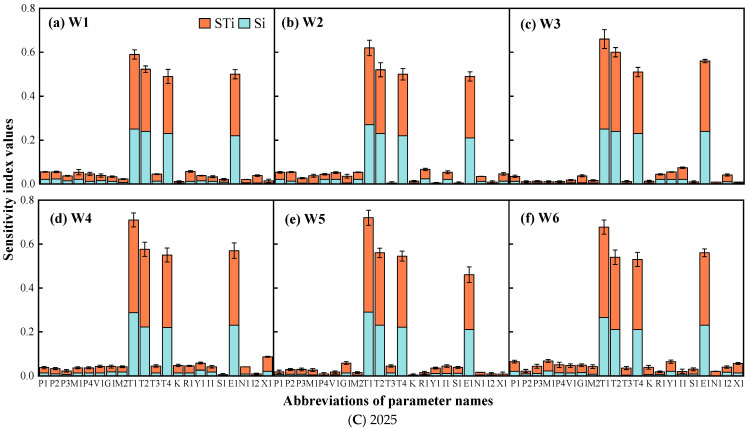
Sensitivity index values of EFAST method for calculating wheat WAGT. W1: the SMC of 9.15% throughout the wheat season; W2: 13.07% SMC during heading and grain filling periods and 9.15% SMC during the other periods; W3: 5.23% SMC during heading and grain filling periods and 9.15% SMC during the other periods; W4: 5.23% SMC during greening and jointing periods and 9.15% SMC during the other periods; W5: 13.07% SMC during greening and jointing periods and 9.15% SMC during the other periods; W6: 13.07% SMC during greening, jointing, and heading periods and 9.15% SMC during the other periods.

The sensitivity distribution of WAGT to the model parameters is shown in Figure 3. In both growing seasons, the parameters with a global sensitivity index greater than 0.10 (influential parameters) were T4, E1, T2, and T1. The sensitivity index of T4 was 0.243~0.365, that of E1 was 0.243~0.393, and the indices for T2 and T1 were 0.257~0.45 and 0.36~0.45, respectively, while the global sensitivity indices of the other parameters were all less than 0.1. The influential parameters were consistent with those obtained using the Morris method.

#### 2.2.2. EFAST Results for Wheat Yield

**Figure 4:**
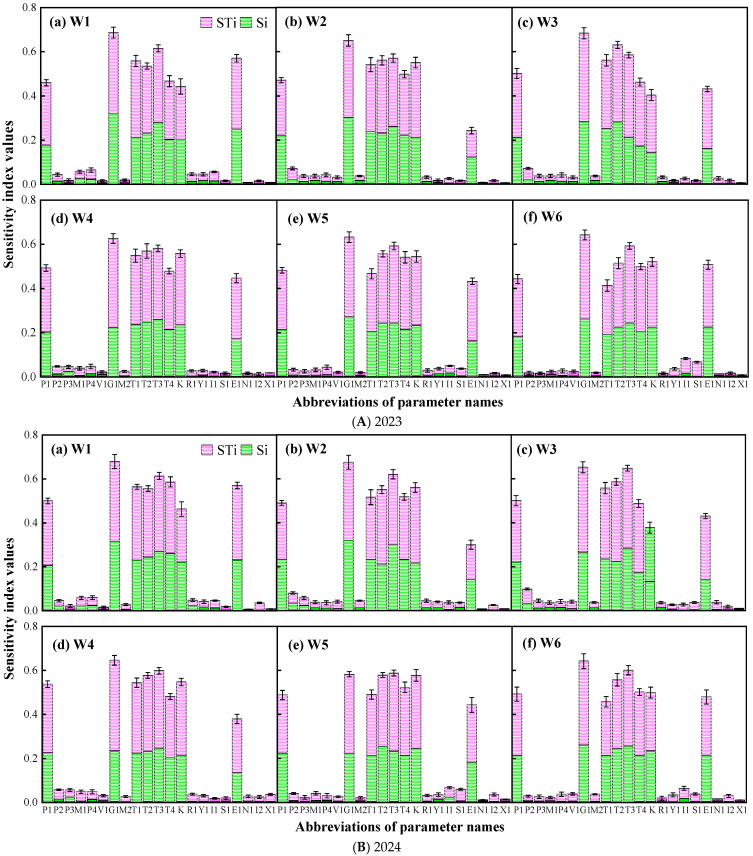

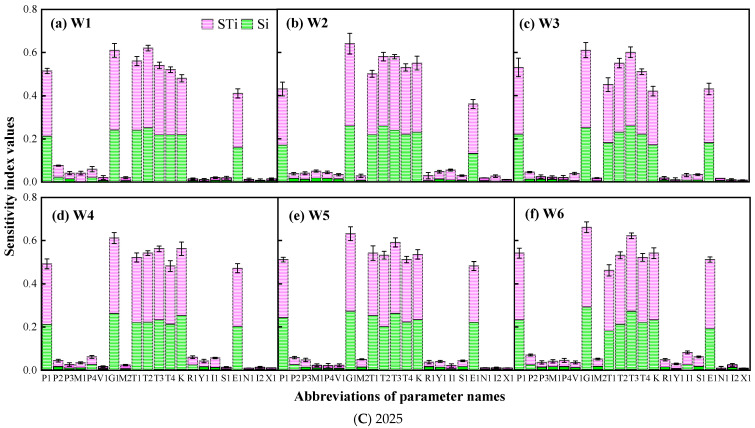
Sensitivity index of EFAST method for calculating wheat yield. W1: the SMC of 9.15% throughout the wheat season; W2: 13.07% SMC during heading and grain filling periods and 9.15% SMC during the other periods; W3: 5.23% SMC during heading and grain filling periods and 9.15% SMC during the other periods; W4: 5.23% SMC during greening and jointing periods and 9.15% SMC during the other periods; W5: 13.07% SMC during greening and jointing periods and 9.15% SMC during the other periods; W6: 13.07% SMC during greening, jointing, and heading periods and 9.15% SMC during the other periods.

The sensitivity distribution of the wheat yield to the model parameters is shown in Figure 4. The parameters with a global sensitivity index greater than 0.10 for the six different soil moisture levels in the three growth seasons were K, T4, P1, T2, E1, G1, T1, and T3. The sensitivity index values were 0.241~0.34 (K), 0.265~0.327 (T4), 0.25~0.29 (P1), 0.291~0.35 (T2), 0.12~0.320 (E1), 0.36~0.402 (G1), 0.221~0.348 (T1), and 0.31~0.372 (T3). The global sensitivity indices of the remaining parameters were all less than 0.1. The influential parameters were consistent with those obtained by the Morris method.

### 2.3. Consistency Test of SA

Consistency tests were conducted on the SA results for yield and WAGT obtained using the Morris method at different soil moisture levels, with all corresponding Top-down concordance coefficient (TDCC) values being greater than or equal to 0.706 and all *p*-values less than 0.01 (Table 1). Similarly, the SA results obtained using the EFAST method at different soil moisture levels consistently yielded TDCC values above 0.658, with all *p*-values less than 0.01 (Table 2). Although these TDCC values were slightly smaller, the results were consistent with those obtained using the Morris method.

### 2.4. Results of Parameter Optimization and Model Evaluation

**Figure 5:**
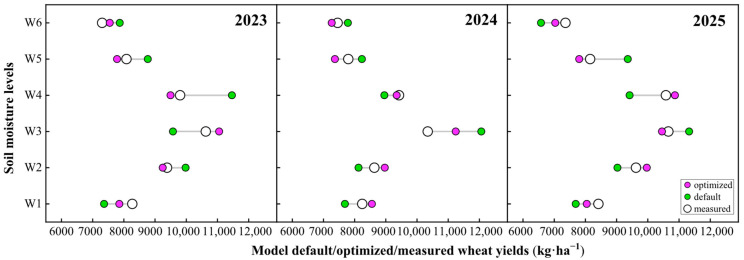
Comparison of default, optimized, and measured wheat yields across soil moisture levels from 2023 to 2025. W1: the SMC of 9.15% throughout the wheat season; W2: 13.07% SMC during heading and grain filling periods and 9.15% SMC during the other periods; W3: 5.23% SMC during heading and grain filling periods and 9.15% SMC during the other periods; W4: 5.23% SMC during greening and jointing periods and 9.15% SMC during the other periods; W5: 13.07% SMC during greening and jointing periods and 9.15% SMC during the other periods; W6: 13.07% SMC during greening, jointing, and heading periods and 9.15% SMC during the other periods.

After optimizing the eight parameters (K, T4, P1, T2, E1, G1, T1, and T3) that strongly influence the yield in the APSIM-Wheat model, the fit between the simulated and observed yields improved across different soil moisture levels. Both sets of values exhibited high consistency in trend and magnitude (Figure 5). The coefficient of determination (R^2^) of the optimized model was 0.877~0.974, index of agreement (d-index) was 0.941~0.995, the root mean square error (RMSE) was 319.45~642.69 kg·ha^–1^, and the mean absolute error (MAE) was 314.69~473.21 kg·ha^–1^. Meanwhile, the residual standard deviation ratio (RSR) was 0.68~0.93, and the Nash–Sutcliffe efficiency (NSE) was 0.26~0.57. Overall, the model performance improved from “Unacceptable” to “Good/Excellent” (Table 3).

## 3. Discussion

The SA can identify the influential parameters of a model from its numerous parameters. Through this process, the uncertainty associated with the model can be decreased [30]. This study found that the simulated values of WAGT and yield of winter wheat were influenced by several parameters of the APSIM-Wheat model. At different soil moisture levels, the parameter T1 exerted the greatest influence on WAGT, which might be attributed to the predominance of vegetative growth during the early stage, thereby establishing a solid foundation for subsequent reproductive development in wheat. At this stage of growth, increased temperatures could boost the photosynthetic ability of winter wheat [31], consequently enhancing the accumulation and partitioning of WAGT. The parameters T3 and G1 had a more pronounced effect on yield. Specifically, T3 significantly impacted grain formation by improving photosynthetic efficiency and leaf physiological status, which supported faster grain filling and higher final yield [32]. Additionally, yield was sensitive to P1 and K. The higher K values might increase the canopy’s utilization of solar radiation, raise photosynthetic rates, accelerate grain filling, and ultimately favor grain formation and yield [33]. The WAGT and yield were highly sensitive to T1, T2, T4, and E1. The increase in temperature during the critical developmental stages of wheat growth significantly impacted WAGT formation and final yield. Appropriate increase in temperature could reduce the dormancy period of wheat, extend its effective growing period, and consequently enhance yield [34]. The parameter T1 affected the duration of the wheat jointing and the overall length of the growing period and accelerated wheat growth and development, leading to a shortened spike differentiation period [35]. The parameter T2 improved wheat growth rate and nitrogen uptake efficiency, thereby supporting faster grain filling and higher yield [36,37]. The parameter T4 influenced the grain filling rate and the final weight of the grains. The parameter E1 significantly influenced photosynthesis in wheat at different growth stages, consequently affecting WAGT and yield. Research indicated that applying appropriate irrigation during critical growth stages, such as jointing and flowering periods, enhanced the net photosynthetic rate and yield of wheat [38]. Studies have shown that changes in the accumulated temperature also affected wheat respiration and physiological indicators, thereby affecting WAGT accumulation [39]. Under low-temperature stress, the synthesis parameters of wheat leaves and endogenous hormone levels significantly decreased [40]. Warming treatment accelerated the processes of greening, jointing, flowering, and maturation in wheat. Meanwhile, the leaf area index and WAGT significantly increased [36]. These results indicated that by reasonably managing the temperature, the yield and productivity of winter wheat could be effectively enhanced. This was consistent with the findings of the present study.

Before applying crop models to specific regions, detailed parameter optimization and applicability validation are crucial [7]. Because different regions may have unique climate conditions, soil types, and management practices, all of which can affect crop growth and model performance. This study identified eight key parameters (K, T4, P1, T2, E1, G1, T1, and T3) that significantly influence winter wheat yield at different soil moisture levels and optimized these influential parameters based on the analysis results. Compared to the non-optimized version, the optimized model exhibited the greater values of r^2^, d-index, and NSE, and the smaller values of RMSE, MAE, and RSR, the metrics used to evaluate the model. The evaluation results indicated that the model’s performance improved from the previous “Unacceptable” level to a “Good/Excellent” standard. The findings further underscored the necessity of parameter optimization when applying the APSIM-Wheat model in specific regions, aiming to minimize uncertainty to the greatest extent [41]. Sexton et al. [42] evaluated the application of the MCMC method in parameter estimation for the APSIM model and found that optimizing parameters via MCMC significantly enhanced the model performance to simulate biomass and yield. Similarly, Akhavizadegan et al. [22], through a computational case study of 25 environments in the U.S. Corn Belt using the APSIM model, confirmed that such parameter optimization improved the model’s yield prediction performance. Collectively, these studies supported the conclusions of the present research.

As the consistency tests of the SA results conducted across the different soil moisture levels shown, the TDCC values were close to 1 and the *p* values were less than 0.01, an indication of highly significant consistency among the SA results across different soil moisture levels. Additionally, the Morris method, which comprehensively incorporated both μ* and σ, produced results consistent with those of the EFAST method. Both approaches identified the same influential parameters and produced similar results (TDCC > 0.658, *p* < 0.01), suggesting that these methods could be used interchangeably. In addition, the computational times required for the sensitivity analyses using Morris and EFAST methods were not the same. For example, conducting EFAST analysis on 21 selected APSIM-Wheat model parameters using a laptop (Intel i5-8250U CPU 1.8 GHz, 8 GB RAM) took approximately 35 h under the same settings. After filtering out non-influential parameters, the computation time was reduced to around 10 h. In contrast, the Morris method took only 3.5 h to complete. As the application of the Morris method was not computationally expensive and the results agreed well with those from the EFAST method, it is suggested that a Morris SA be performed under the local environmental conditions before model simulations.

## 4. Materials and Methods

### 4.1. Site Description

The experiment was conducted at the Huaxing Farm Irrigation Zone Test Base within the Changji National Agricultural Science and Technology Park (87°18′0″ E, 44°12′6″ N; altitude 578 m) in the Xinjiang Autonomous Region, China, during the years 2023 through 2025. The region has a temperate continental arid climate with an annual precipitation of approximately 190 mm. It receives minimal rainfall and abundant solar energy, leading to an average annual temperature of 6.5 °C. The predominant soil type is sandy loam, with a groundwater depth exceeding 10 m. The average bulk density of the 60 cm soil layer is 1.62 g·cm^−^^3^, while the field capacity (FC) is 26.15%. Soil characteristics of the experimental site are presented in Table 4.

### 4.2. Field Experiment

**Figure 6:**
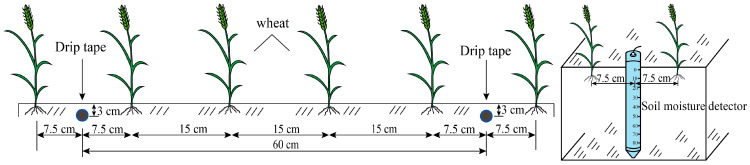
Layout of planting, drip irrigation belts, and soil moisture detectors in the experimental plot.

The wheat variety used was ‘XinDong 22’, with sowing for all three seasons from 2023 to 2025 occurring in mid-September and harvesting taking place in early July. The layout of experimental plots, drip irrigation belts, and soil moisture detectors is shown in Figure 6. Shallow buried drip irrigation was used for irrigation, and the water volume was controlled using water meter and bypass valve. The fertilization practices for each treatment were consistent with local field management standards. Irrigation was managed by controlling the upper and lower soil moisture limits, with the upper limit set at FC (FC and the lower limits as specified in Table 5). In Table 5, the soil moisture content (SMC) was calculated as the maximum soil moisture level for irrigation (FC) minus the minimum soil moisture level for irrigation (50%, 65%, or 80% of FC). The purpose of selecting different soil moisture lower levels was to investigate how various water management strategies implemented at different growth stages of winter wheat affected its growth and development and to further analyze the underlying causes and characteristics of these changes. This decision was based on preliminary surveys and literature reviews, which revealed that a 65% FC was the most commonly used lower limit for winter wheat irrigation. Therefore, this value was adjusted by ±15%, resulting in the inclusion of 50% and 80% FC. Furthermore, based on practical experience, soil moisture levels below 50% FC could lead to crop wilting; thus, we avoided setting it lower than 50% FC to prevent prolonged wilting that could not be reversed.

Soil moisture was continuously monitored in real time using sensors equipped for continuous measurement. When soil moisture fell to the preset lower irrigation level, irrigation was applied according to a single irrigation quota and regulated to maintain soil moisture below the upper limit (FC). The experimental design was informed by the study of Kuang et al. [43]. The study utilized a completely randomized block design, incorporating six irrigation treatments labeled W1 to W6, with W1 serving as the control group. Each treatment was repeated thrice, resulting in 18 plots, each with an area of 168 m^2^.

### 4.3. APSIM-Wheat Model

This study used APSIM version 7.10, primarily involving the wheat module, namely APSIM-Wheat. The primary driving factors of the model are conventional meteorological data, namely daily maximum and minimum temperatures, precipitation, and solar radiation, all sourced from the Changji experimental field weather station, located approximately 100 m from the test site. More information and code related to the model can be found on the official website: http://www.apsim.info/ (accessed on 20 May 2025).

APSIM is a mechanistic model developed by the Australian Agricultural Production Systems Unit (APSRU) to simulate the main components of agricultural production systems [44,45]. The model employs a central engine to seamlessly integrate and manage the crop, management, soil, and meteorological modules in a modular manner. The “plugin” architecture of the model allows all the modules to be integrated into the base model. This promotes flexibility in management practices. It can simulate crop growth dynamics, yield formation, soil moisture, and temperatures. These simulations are applicable to a combination of various climate, variety, soil, and management factors The changes in each module focus on the soil, with weather variations and field management adjustments causing changes in soil water and nutrient limitation conditions. Simultaneously, daily meteorological data from weather datasets serve as driving variables to control the growth and development of wheat through dynamic simulation with a daily step.

### 4.4. Global SA Methods

#### 4.4.1. Morris Method

The Morris method used in this study was initially introduced by Morris in 1991 and later refined by Campolongo et al. [27,46]. The method is described as follows:

(1) $$R_{i}\left(x_{1},x_{2},\ldots ,x_{n},\Delta \right)\;=\;\frac{y\left(x_{1},x_{2},\ldots ,x_{i-1},x_{i}+\Delta ,x_{i+1},\ldots ,x_{n}\right)\;-\;y\left(x_{1},x_{2},\ldots ,x_{n}\right)}{\Delta }$$

Let *X* = (*x*_1_, *x*_2_, *…*, *x_n_*), where *R_i_* (*X*, Δ) denotes the sensitivity index for parameter *i*, *y*(*X*) represents the model output, and *X* is the n-dimensional vector of parameters to be analyzed. Δ is a value ranging between 1 / (p − 1) and 1 − 1 / (p − 1). *p* is the parameter level.

Given the inherent randomness of the Morris method, errors are prone to occur during a single random sampling and randomization process, which necessitated *t* repetitions. Finally, μ* and *σ* for each parameter *R_i_* were calculated, with the model being executed *t*(*n* + 1) times. The μ* reflects the sensitivity of a parameter to the output variable—the larger the value, the greater the sensitivity. The *σ* value indicates the strength of the interactions between parameters or the nonlinear effects of the parameter—the greater the value, the stronger the parameter interaction.

This study characterized the parameters where μ*_average_ < μ* < μ*_max_ as influential parameters [47].

#### 4.4.2. EFAST Method

The EFAST method was used to address the issue of slow simulation speed in complex crop growth models [26]. A brief introduction to this algorithm is provided below:

The total variance of the model is decomposed into


(2) $$\;V=\sum_{1⩽i⩽m}\;V_{i}+\sum_{1⩽i⩽j⩽m}\;V_{i,j}+\sum_{1⩽i⩽j⩽k⩽m}\;V_{i,j,k}+\ldots +V_{1,2,\ldots ,m}$$

In the equation, *V_i_*, *j* represents the model variance caused by parameter *x_i_* through parameter *x_j_*; *V_i_*, *_j_*, *_k_* represents the model variance caused by parameter *x_i_* through parameters *x_j_* and *x_k_*; and *V*_1_, _2_, …, *_m_* represents the model variance caused by parameter *x_i_* through interactions with the remaining *m* − 1 parameters. After normalization, the first-order sensitivity index (*S_i_*) of parameter *x_i_* is


(3) $$S_{i}=\frac{V_{i}}{V}$$

The global sensitivity index (*S_Ti_*) is


(4) $$S_{\mathrm{Ti}}=\frac{V\;{-V}_{-i}}{V}$$

In the equation, *V_−i_* is the total variance of all variables except *x_i_*.

This study established *S_i_* > 0.05 and *S_Ti_* > 0.10 (*S_i_* + *S_Ti_* > 0.15) as the criteria for identifying influential parameters [48].

### 4.5. Parameter Selection and SA Plan

This study used data on weather, soil, and crop management collected from field experiments. The selection of the APSIM-Wheat model parameters was based on the study by Zhao et al. [49] and the model documentation [50]. During the sensitivity analyses, the range of each parameter value was changed by ±50% from the initial value, as illustrated in Table 6. The sensitivity of simulated wheat yield and WAGT to the 21 parameters of the APSIM-Wheat model was evaluated using Morris and EFAST methods.

Global SA experiments were implemented on SimLab2.2, a specialized software for SA. The APSIM-Wheat model underwent batch simulations using the apsimx package in R 4.3.3, facilitated by Rstudio (Boston, MA, USA). The procedural steps were as follows:

(1) The range of model parameters was defined in SimLab2.2 and a uniform distribution was assumed for these parameters.
(2) The input parameters were sampled. The Morris method required setting *t* = 10 (repetitions), *n* = 21 (parameters) × 3 (years), and sampling a total of 640 groups of 10 × (21 × 3 + 1). The EFAST method generated 4410 sets of 21 (parameters) × 3 (years) × 70 (times). The EFAST method deemed valid when the number of samples exceeded 65 times the number of parameters; accordingly, this study adopted a sampling size 70 times greater than the number of parameters.
(3) The R language was used to modify parameters, run in batches, and organize the results for the APSIM-Wheat model.
(4) The batch-processed simulation results were organized into a format recognizable by SimLab2.2, inputted into the software for analysis, and the SA results were obtained.

### 4.6. Consistency Test of SA Results

The consistency of the SA results across different soil moisture levels, obtained using Morris and EFAST methods, was evaluated using the TDCC [51]. The TDCC was calculated by assigning Savage scores to each parameter, which emphasized the importance of influential parameters while reducing the significance of those with little impact. The savage score of the ranked matrix is calculated as follows:

(5) $$\mathrm{ss}(S_{\mathrm{ij}})=\sum_{i=r(S_{\mathrm{ij}})}^{n}\;1/i$$

where *S_ij_* represents the sensitivity measure of parameter *x_i_* and repetition *R*_j_. *R_Sij_*, *i* = 1, 2, …, *n*, represents the rank assigned to the sensitivity measure corresponding to *R_j_.* Rank 1 is assigned to the parameter with the largest *S_ij_* value, rank 2 to the second largest, and so on.

For all parameters *x_i_* and repetitions *R_j_*, the formula that this study used for calculating TDCC is as follows:

(6) $$\begin{array}{c} C_{T\;}=\left\{\sum_{i=1}^{n}\;{\left[\sum_{j=1}^{m}\;\mathrm{ss}\left(S_{\mathrm{ij}}\right)\right]}^{2}-{\;m}^{2}n\right\}/\\ \left\{m^{2}\left(n-\sum_{i=1}^{n}\;1/i\right)\right\} \end{array}$$

where *m* represents the number of repetitions for the SA, corresponding to different soil moisture levels; *n* represents the 21 parameters of the APSIM-Wheat model to be analyzed.

The significance *p*-value of the TDCC is calculated from the statistical value *T* as follows:

(7) $$T=m(n\;-\;1)C_{T}$$

where *T* represents a *χ^2^* distribution with *n* − 1 degrees of freedom.

The closer the TDCC value was to 1, the better the consistency. If the *p*-value of the TDCC test was less than 0.05, the SA results were considered to have significant consistency [52].

### 4.7. Parameter Optimization and Model Evaluation

Parameter optimization and model evaluation were conducted using the comprehensive data on soil and crops measured during 2023~2025. The data on the initial soil moisture and soil salinity were obtained from field measurements taken prior to sowing. Other crop parameters for winter wheat were refined as recommended in the APSIM-Wheat model documentation.

Parameter optimization was conducted using a Bayesian Markov Chain Monte Carlo (MCMC) method. Assuming the parameters were situated within a probabilistic space and modeled using a normal distribution. The application of Bayes’ theorem (posterior theorem) is as follows:

(8) $$P(ϴ|O)=\frac{P(O|ϴ)\;\times \;P(ϴ)}{P(O)}=L[ϴ|O]\;\times \;P(ϴ)$$

where *P*(*O*/*θ*) denotes the probability of the observations given the parameters. *P*(*θ*) refers to the prior probability of the parameters. *P*(*O*) denotes the probability of the observed data. *P*(*θ*/*O*) represents the posterior probability distribution of the optimal parameters after incorporating the observed values. *L*[*θ*/*O*] denotes the likelihood function.


(9) $$L\left[{ϴ}_{i}|O\right]=\prod_{j=1}^{M}\;\frac{1}{\sqrt{2\pi \sigma_{o}^{2}}}\exp\left(-\frac{{\left(\bar{O}\;-\;P\left({ϴ}_{i}\right)\right)}^{2}}{2\sigma_{o}^{2}}\right)$$

Here, $\bar{O}$ indicates the mean of observed values.

The likelihood function followed a Gaussian (normal) distribution. As indicated by the equation above, a higher posterior probability corresponding to a larger likelihood value indicated that the simulation was more accurate. In this study, the Markov Chain Monte Carlo method was implemented using the R programming language.

The agreement between observed yield values and model simulations was assessed using R^2^, RMSE, d-index, MAE, NSE, and RSR. Models with R^2^ and d values closer to 1 and RMSE and MAE values closer to 0 were considered to have better conformity between simulated and observed yields [53]. Additionally, model evaluation was conducted using NSE and RSR, with grading criteria based on Moriasi and Ai et al. [53,54].

### 4.8. Statistical Analysis

Data on winter wheat yield and WAGT, soil, SA, and other related variables were preliminarily organized, summarized, and calculated using Microsoft Excel 2021 (Microsoft Corp., Redmond, WA, USA). Correlation analysis between the treatments was performed using DPS 7.05 (Data Processing System, Hangzhou Ruifeng Information Technology Co., Ltd., Hangzhou, China). Multiple comparisons between the treatments were conducted using Duncan’s method (*α* = 0.05). Plots were generated using Origin 9.0 (Origin Lab Corp., Northampton. MA, USA) software.

## 5. Conclusions

In this study, we conducted global SA of the APSIM-Wheat model in the dryland agricultural region of Changji, located in northern Xinjiang, China. A total of 21 model parameters were evaluated using Morris and EFAST methods. The results from both methods were highly consistent, indicating that only 19% to 38% of the parameters significantly impacted on wheat yield and WAGT. The sensitivity indices for different soil moisture levels showed highly significant consistency. Then, the wheat model only with the significantly influential parameters was evaluated using three years’ data on yield and WAGT. The model’s simulated yields were in good agreement with the measured yields, indicating that the APSIM-Wheat model could accurately simulate the impacts of various management and environmental factors on wheat productions systems in northern Xinjiang and other arid regions of China with similar climatic conditions. Although, the SA of the APSIM-Wheat model and its parameter estimation were conducted under the specific climate, soil, and crop management conditions of Changji, China, the model could be applicable to other similar arid regions such as Central Asia and the Middle East. This study focused exclusively on the “Xindong 22” winter wheat variety in Changji, Xinjiang, China. Due to the limited diversity of cultivars and the region’s unique climatic conditions, it is difficult to comprehensively evaluate the adaptability of the APSIM-Wheat model under different climate conditions. In the future, it will be necessary to establish experimental sites in various representative arid regions and incorporate multi-variety comparison trials to systematically investigate the regional heterogeneity of this model within arid agricultural systems.

## Figures and Tables

**Table 1:** Consistency test of the SA results using the Morris method at different soil moisture levels (W1, W2, W3, W4, W5, W6).

Years	Items	W1		W2		W3		W4		W5		W6	
		TDCC	*p*	TDCC	*p*	TDCC	*p*	TDCC	*p*	TDCC	*p*	TDCC	*p*
2023	WAGT	0.775	<0.01	0.758	<0.01	0.847	<0.01	0.738	<0.01	0.725	<0.01	0.712	<0.01
	Yield	0.786	<0.01	0.723	<0.01	0.768	<0.01	0.747	<0.01	0.708	<0.01	0.756	<0.01
2024	WAGT	0.834	<0.01	0.766	<0.01	0.863	<0.01	0.767	<0.01	0.832	<0.01	0.789	<0.01
	Yield	0.778	<0.01	0.709	<0.01	0.787	<0.01	0.872	<0.01	0.756	<0.01	0.862	<0.01
2025	WAGT	0.767	<0.01	0.722	<0.01	0.885	<0.01	0.745	<0.01	0.824	<0.01	0.752	<0.01
	Yield	0.824	<0.01	0.706	<0.01	0.759	<0.01	0.854	<0.01	0.732	<0.01	0.816	<0.01

WAGT: aboveground total dry matter; TDCC: top-down concordance coefficient. W1: the SMC of 9.15% throughout the wheat season; W2: 13.07% SMC during heading and grain filling periods and 9.15% SMC during the other periods; W3: 5.23% SMC during heading and grain filling periods and 9.15% SMC during the other periods; W4: 5.23% SMC during greening and jointing periods and 9.15% SMC during the other periods; W5: 13.07% SMC during greening and jointing periods and 9.15% SMC during the other periods; W6: 13.07% SMC during greening, jointing, and heading periods and 9.15% SMC during the other periods. A *p*-value of less than 0.01 is considered highly significant.

**Table 2:** Consistency test of the SA results using the EFAST method at different soil moisture levels (W1, W2, W3, W4, W5, W6).

Years	Items	W1		W2		W3		W4		W5		W6	
		TDCC	*p*	TDCC	*p*	TDCC	*p*	TDCC	*p*	TDCC	*p*	TDCC	*p*
2023	WAGT	0.821	<0.01	0.801	<0.01	0.883	<0.01	0.844	<0.01	0.763	<0.01	0.892	<0.01
	Yield	0.746	<0.01	0.658	<0.01	0.757	<0.01	0.665	<0.01	0.754	<0.01	0.767	<0.01
2024	WAGT	0.808	<0.01	0.726	<0.01	0.698	<0.01	0.753	<0.01	0.714	<0.01	0.722	<0.01
	Yield	0.738	<0.01	0.665	<0.01	0.734	<0.01	0.711	<0.01	0.765	<0.01	0.752	<0.01
2025	WAGT	0.767	<0.01	0.785	<0.01	0.776	<0.01	0.816	<0.01	0.763	<0.01	0.843	<0.01
	Yield	0.676	<0.01	0.661	<0.01	0.712	<0.01	0.704	<0.01	0.697	<0.01	0.726	<0.01

WAGT: aboveground total dry matter, and TDCC: Top-down concordance coefficient. W1: the SMC of 9.15% throughout the wheat season; W2: 13.07% SMC during heading and grain filling periods and 9.15% SMC during the other periods; W3: 5.23% SMC during heading and grain filling periods and 9.15% SMC during the other periods; W4: 5.23% SMC during greening and jointing periods and 9.15% SMC during the other periods; W5: 13.07% SMC during greening and jointing periods and 9.15% SMC during the other periods; W6: 13.07% SMC during greening, jointing, and heading periods and 9.15% SMC during the other periods. A *p*-value of less than 0.01 is considered highly significant.

**Table 3:** Results of model evaluation on wheat yields from 2023 to 2025.

Year	Parameter Set	R^2^	d-Index	RMSE (kg·ha^–1^)	MAE (kg·ha^–1^)	NSE	RSR	Grade
2023	Default	0.526	0.822	985.28	910.32	0.24	0.85	Unacceptable
	Optimized	0.877	0.941	642.69	473.21	0.68	0.57	Good
2024	Default	0.768	0.885	821.07	669.80	0.30	0.82	Unacceptable
	Optimized	0.955	0.961	454.83	373.34	0.79	0.46	Excellent
2025	Default	0.666	0.918	889.55	856.61	0.49	0.70	Unacceptable
	Optimized	0.974	0.995	319.45	314.69	0.93	0.26	Excellent

**Table 4:** Soil properties of winter wheat experimental site in Changji, Xinjiang.

Soil Layer cm	Organic Matter g·kg^−1^	Available P mg·kg^−1^	Available K mg·kg^−1^	Nitrate Nitrogen mg·kg^−1^	Ammonium Nitrogen mg·kg^−1^	Total N g·kg^−1^	Total P g·kg^−1^	Total K g·kg^−1^	pH
0~20	15.04	6.54	339.86	13.32	4.50	0.56	1.12	18.54	7.60
20~40	12.23	5.61	203.43	10.65	4.86	0.42	1.01	16.52	7.81
40~60	9.12	4.12	160.12	12.43	4.65	0.33	0.89	15.16	8.03

**Table 5:** Values of the lower soil moisture limit as percentage of the field capacity and the corresponding soil moisture content (%) by winter wheat growth phase under various soil moisture treatment levels (W1, W2, W3, W4, W5, W6).

Treatment	Seedling Period	Wintering Period	Greening Period	Jointing Period	Heading Period	Grain Filling Period	Maturity Period
W1	65	65	65	65	65	65	65
	9.15%	9.15%	9.15%	9.15%	9.15%	9.15%	9.15%
W2	65	65	65	65	50	50	65
	9.15%	9.15%	9.15%	9.15%	13.07%	13.07%	9.15%
W3	65	65	65	65	80	80	65
	9.15%	9.15%	9.15%	9.15%	5.23%	5.23%	9.15%
W4	65	65	80	80	65	65	65
	9.15%	9.15%	5.23%	5.23%	9.15%	9.15%	9.15%
W5	65	65	50	50	65	65	65
	9.15%	9.15%	13.07%	13.07%	9.15%	9.15%	9.15%
W6	65	65	50	50	50	65	65
	9.15%	9.15%	13.07%	13.07%	13.07%	9.15%	9.15%

**Table 6:** The upper and lower bounds of the APSIM-Wheat model parameters.

Abbreviation	Definition	Unit	Lower Bound	Upper Bound
P1	potential daily grain filling rate during the grain filling period	g·grain^−1^·d^−1^	0.001	0.004
P2	potential daily grain filling rate from flowering to grain filling stage	g·grain^−1^·d^−1^	0.0005	0.0015
P3	daily potential grain nitrogen accumulation rate	g·grain^−1^·d^−1^	0.0000275	0.0000825
M1	lower limit of daily nitrogen accumulation rate in grains	g·grain^−1^·d^−1^	0.0000075	0.0000225
P4	crop photoperiod sensitivity index	/	0	5
V1	crop vernalization sensitivity index	/	0	5
G1	number of grains per unit stem	grain·g^−1^	10	40
M2	maximum grain weight per plant	g	0.02	0.06
T1	accumulated temperature from seedling to jointing stage	°C·d	200	600
T2	accumulated temperature from jointing to flowering period	°C·d	250	800
T3	accumulated temperature from flowering to grain filling period	°C·d	60	180
T4	accumulated temperature from grain filling to maturity	°C·d	200	900
K	extinction coefficient	/	0	1
R1	light energy utilization rate	g·MJ^−1^	1.116	1.364
Y1	maximum specific leaf area	mm^2^·g^−1^	22,000	45,000
I1	leaf area at the beginning of the plant	mm^2^	100	300
S1	slope of water stress in photosynthetic leaf aging	/	0.05	0.15
E1	crop water demand	/	0.75	2.25
N1	multiple effects of nitrogen deficiency on photosynthesis	/	0.75	2.25
I2	maximum leaf area index of aging caused by shading	m^2^·m^–2^	3.5	10.5
X1	daily average temperature affects the grouting rate	/	0	1

## Data Availability

The data presented in this study are available on request from the corresponding author. The data are not publicly available due to the policies and confidentiality agreements adhered to in laboratory; therefore, we regretfully cannot furnish the raw data.
